# The prevalence of cardiovascular disease and antidiabetes treatment characteristics among a large type 2 diabetes population in the United States

**DOI:** 10.1002/edm2.76

**Published:** 2019-05-22

**Authors:** Wayne Weng, Ye Tian, Sheldon X. Kong, Rahul Ganguly, Malene Hersloev, Jason Brett, Todd Hobbs

**Affiliations:** ^1^ Novo Nordisk Inc. Plainsboro New Jersey

**Keywords:** atherosclerotic cardiovascular disease, epidemiology, GLP‐1RA, real‐world, SGLT‐2i, type 2 diabetes mellitus

## Abstract

**Objectives:**

The purpose of this study was to assess atherosclerotic cardiovascular disease (ASCVD) prevalence, antidiabetes medication usage and physician specialty encounters among individuals with type 2 diabetes mellitus (T2DM) in the United States during 2015.

**Design:**

Retrospective, cross‐sectional analysis.

**Patients:**

Adults with T2DM in a large US administrative claims database. Patients were divided into ASCVD and non‐ASCVD groups. Subgroup analyses were conducted for three age groups (18‐44, 45‐64 and 65+ years).

**Results:**

Of 1 202 596 patients with T2DM, 45.2% had established ASCVD. About 40% of T2DM patients with ASCVD had visited a cardiologist during 2015, compared to 11% in the non‐ASCVD group. The use of glucagon‐like peptide‐1 receptor agonists (GLP‐1RAs) and sodium‐glucose co‐transporter 2 inhibitors (SGLT‐2is) was low overall (<12%), and even lower in the ASCVD group (<9%). The prevalence of ASCVD was 15%, 36% and 71% in the 18‐44, 45‐64 and 65+ year age groups, respectively. GLP‐1RA and SGLT‐2i use was ≤5% in the 65+ subgroup, regardless of ASCVD status.

**Conclusions:**

These real‐world data showed a high prevalence of ASCVD among T2DM patients, and confirmed, as a baseline assessment, low use of GLP‐1RAs and SGLT‐2is in these at‐risk patients prior to the 2017 American Diabetes Association guidelines recommending use of agents with proven cardiovascular benefits.

## 1 INTRODUCTION

Cardiovascular disease (CVD) is one of the most prevalent comorbidities of type 2 diabetes mellitus (T2DM)^1^, ^2^, ^3^ and the primary cause of death in patients with T2DM.^4^ Effective glucose lowering alone is not consistently linked with a clinically relevant impact on lowering risk of adverse cardiovascular (CV) outcomes.^5^, ^6^, ^7^ Identification of clinically effective and cost‐efficient strategies for the co‐management of T2DM and CVD continues to be an important goal to improve health and contain healthcare expenditures.

In recent years, large cardiovascular outcomes trials (CVOTs) have demonstrated CV benefits with glucagon‐like peptide‐1 receptor agonists (GLP‐1RA) and sodium‐glucose co‐transporter‐2 inhibitor (SGLT2is). The first such studies to be published were the EMPA‐REG‐OUTCOME trial in 2015^8^ and the Liraglutide Effect and Action in Diabetes: Evaluation of Cardiovascular Outcome Results (LEADER) trial in 2016.^9^ In the EMPA‐REG‐OUTCOME study, patients with established, stable CV disease treated with the SGLT2i empagliflozin had a lower rate of the primary composite outcome (death from cardiovascular causes, nonfatal myocardial infarction or nonfatal stroke) than patients receiving placebo, as well as significantly lower rates of death from cardiovascular causes, hospitalization for heart failure and death from any cause.^8^ In the LEADER trial, patients with T2DM and concomitant CV disease or at high CV risk treated with the GLP‐1RA liraglutide had a lower rate of the primary composite outcome (first occurrence of cardiovascular death, nonfatal myocardial infarction or nonfatal stroke in the time‐to‐event analysis), and lower risks of death from cardiovascular causes, death from any cause and microvascular events than did those receiving placebo.^9^

Based on the results of these two studies, the American Diabetes Association (ADA), in their 2017 Standards of Medical Care in Diabetes,^4^ incorporated a specific recommendation to consider empagliflozin or liraglutide in patients with established atherosclerotic CVD (ASCVD) to reduce the risk of mortality. Subsequently, the results of additional, positive CVOTs became available and the 2018 ADA Standards of Medical Care includes the recommendation “to incorporate an agent with strong evidence for cardiovascular risk reduction, especially those with proven benefit on both major adverse cardiovascular events and cardiovascular death.”^10^ More recently, the ADA and EASD issued a Consensus Report on the Management of Hyperglycemia in Type 2 Diabetes,^11^ in which SGLT2is or GLP‐1 receptor agonists with proven cardiovascular benefit are recommended for patients with T2DM who have established ASCVD.

The impact of the updated diabetes guidelines regarding GLP‐1RA and SGLT2i use in at‐risk patients in real‐world clinical practice will be of interest in the coming years. A recent study used the electronic health record system at Cleveland Clinic (Ohio and Florida) to create a cross‐sectional summary of patients with T2DM and CVD in 2016 (ie the year prior to release of the 2017 ADA guidelines) to establish a baseline of real‐world treatment patterns in these patients.^12^ Utilization rates of GLP‐1RA and SGLT2i agents were found to be low (<10%) in patients with T2DM, whether with or without established CVD.

The current study used real‐world claims data to determine the prevalence of ASCVD among patients with T2DM and to assess antidiabetes medication usage and healthcare specialty utilization in these high‐risk patients prior to availability of the 2017 ADA guidelines.

## 2 METHODS

### 2.1 Data source and study population

This was a retrospective, cross‐sectional analysis of a large, nationwide US administrative claims database (IBM® Family of MarketScan® Research Databases, formerly Truven Health Analytics MarketScan Databases) using 2015 data. The MarketScan database contains de‐identified, individual healthcare claims data from all US states and is fully compliant with the Health Insurance Portability and Accountability Act of 1996.

Eligible individuals were aged ≥18 years on 1 January 2015 and had an established diagnosis of T2DM before 1 January 2015, defined as ≥2 diagnoses for T2DM, based on international classification of diseases, ninth revision (ICD‐9) codes of 250.x0 or 250.x2 or ICD‐10 codes of E11.xx or ≥1 T2DM diagnosis with ≥1 oral antidiabetes drug (OAD) claim, and no more than 1 T1D diagnosis according to ICD‐9 (250.x1, 250.x3) or ICD‐10 (E10.x) codes. Continuous health plan enrolment with an insurance plan containing both medical and pharmacy benefits between 1 January 2014 and 31 December 2015 was required. The baseline period was defined as 1 January 2014‐31 December 2014, and the study period was defined as 1 January 2015‐31 December 2015.

## 3 Study cohorts—patients with and without established ASCVD

Eligible patients with T2DM were divided into two groups based on the presence (ASCVD group) or absence (non‐ASCVD group) of established ASCVD. To be included in the ASCVD group, ASCVD must have been present prior to 1 January 2015 and was defined based on the ICD‐9/‐10 codes (Table S1), corresponding to ADA 2017 Standards of Medical Care definition of ASCVD: acute coronary syndrome (ACS), history of myocardial infarction (MI), stable or unstable angina pectoris, peripheral arterial disease (PAD) presumed to be of atherosclerotic origin, stroke, transient ischaemic attack (TIA) and coronary or other arterial revascularization.^4^

## 4 Variables of interest

Patient demographics were determined as of 1 January 2015 and included age, sex, geographic region and insurance type. Comorbidities were identified from claims from 2014 and 2015 and were used to determine the Diabetes Complications Severity Index (DCSI) score,^13^ and Charlson Comorbidity Index (CCI) score.^14^ Individual comorbidities of hypertension and dyslipidemia were also recorded, as these were not captured in the definition of ASCVD nor were they components of DCSI or CCI scoring.

Study endpoints during 2015 included claims for any anti‐diabetes medications, use of GLP‐1RA and SGLT2i agents specifically and visits with endocrine or cardiovascular specialists.

### 4.1 Data analysis

This was a descriptive analysis. Population characteristics were measured using counts with percentages for the categorical variables and means with standard deviation (SD) for continuous variables. Subgroup analyses were conducted for three age categories (18‐44, 45‐64 and ≥65 years).

## 5 RESULTS

### 5.1 Study population

There were 16 300 609 individuals in the MarketScan database who had continuous enrolment from 1 January 2014 to 31 December 2015; of these, 13 106 234 were aged ≥18 years in 2015 (index year), and of these, 1 202 596 patients with T2DM were identified who met all other eligibility criteria and comprised the study population. Just under half (45.2%; n = 543 098) of patients had established ASCVD Table 1. The ASCVD group was older than the non‐ASCVD group (mean age, 67 vs 56 years), had a slightly higher percentage of males (52.9% vs 49.2%) and a much greater proportion having Medicare insurance (49.5% vs 16.1%).

**Table 1:** Demographic characteristics of a real‐world 2015 population with type 2 diabetes (N = 1 202 596), stratified by atherosclerotic cardiovascular disease (ASCVD) status

Variable	All patients N = 1 202 596 (100.0%)	By ASCVD status	
		Non‐ASCVD n = 659 498 (54.8%)	ASCVD n = 543 938 (45.2%)
Age, y, mean (SD)	60.9 (12.8)	56.2 (11.3)	66.5 (12.3)
Age category, n (%)			
18‐44 y	110 676 (9.2)	93 646 (14.2)	17 030 (3.1)
45‐64 y	707 272 (58.8)	452 819 (68.7)	254 453 (46.9)
65+ y	384 648 (32.0)	113 033 (17.1)	271 615 (50.0)
Gender, n (%)			
Female	590 874 (49.1)	335 295 (50.8)	255 579 (47.1)
Male	611 722 (50.9)	324 203 (49.2)	287 519 (52.9)
Region of US, n (%)			
North Central	316 215 (26.3)	143 870 (21.8)	172 345 (31.7)
Northeast	235 934 (19.6)	123 048 (18.7)	112 886 (20.8)
South	505 517 (42.0)	297 423 (45.1)	208 094 (38.3)
West	142 568 (11.9)	93 867 (14.2)	48 701 (9.0)
Unknown	2362 (0.2)	1290 (0.2)	1072 (0.2)
Insurance, n (%)			
Commercial	828 065 (68.9)	553 676 (84.0)	274 389 (50.5)
Medicare	374 531 (31.1)	105 822 (16.1)	268 709 (49.5)
ASCVD Diagnosis,^a^ n (%)			
Acute Coronary Syndrome	319 931 (26.6)	–	319 931 (58.9)
Angina pectoris	111 209 (9.3)	–	111 209 (20.5)
Myocardial infarction	89 498 (7.4)	–	89 498 (16.5)
Peripheral arterial disease	294 092 (24.5)	–	294 092 (54.1)
Revascularization	93 365 (7.7)	–	93 365 (17.2)
Stroke	223 736 (18.6)	–	223 736 (41.2)
Transient ischaemic attack	76 790 (6.4)	–	76 790 (14.1)
Comorbidities			
Hypertension, n (%)	950 941 (79.1)	472 299 (71.6)	478 642 (88.1)
Dyslipidemia, n (%)	934 967 (77.8)	484 175 (73.4)	450 792 (83.0)
Diabetes‐related complications,^b^ n (%)			
Retinopathy	145 528 (12.1)	63 101 (9.6)	82 427 (15.2)
Nephropathy	183 043 (15.2)	61 975 (9.4)	121 068 (22.3)
Cerebrovascular	118 557 (9.9)	0 (0.0)	118 557 (21.8)
Cardiovascular^c^	334 933 (27.9)	23 545 (3.6)	311 388 (57.3)
Peripheral vascular^d^	125 519 (10.4)	14 388 (2.2)	111 131 (20.5)
Metabolic	149 080 (12.4)	79 359 (12.0)	69 721 (12.8)
DCSI score, mean (SD)	1.7 (2.0)	0.8 (1.2)	2.7 (2.3)
CCI score, mean (SD)	2.4 (2.1)	1.7 (1.4)	3.3 (2.4)

Abbreviations: ASCVD, atherosclerotic cardiovascular disease; SD, standard deviation.

a As defined by ADA 2017 guidelines. Patients could have more than one.

b Comorbidities included in the Diabetes Complications Severity Index.^13^

c Category includes any cardiovascular complication, not limited to those used to define “ASCVD” (acute coronary syndrome, history of myocardial infarction, angina pectoris, peripheral arterial disease presumed to be of atherosclerotic origin, transient ischaemic attack and coronary or other arterial revascularization).

d Category includes any peripheral vascular disease, not limited to “peripheral arterial disease presumed to be of atherosclerotic origin” which was part of the “ASCVD” definition. Category includes ketoacidosis, hyperosmolar and “other coma.”

The burden of hypertension and dyslipidemia comorbidity was also higher in the ASCVD group compared with the non‐ASCVD group (hypertension: 88.1% vs 71.6%; dyslipidemia 83.0% vs 73.4%).

### 5.2 Antidiabetes drug use patterns

In the total population with T2DM, the majority of patients had claims for OADs only, regardless of ASCVD status Table 2. Among OAD‐only users, most patients were using 1 (56%‐57%) or 2 (30%) OADs.

**Table 2:** Antidiabetes medication treatment patterns stratified by atherosclerotic cardiovascular disease (ASCVD) status

Medication	Non‐ASCVD N = 659 498	ASCVD N = 543 938
OAD only, n (%)	340 485 (77.0)	243 967 (73.6)
1 OAD	189 412 (55.6)	138 907 (56.9)
2 OAD	103 133 (30.3)	73 194 (30.0)
≥3 OAD	47 940 (14.1)	31 866 (13.1)
Insulin ± OAD, n (%)	61 278 (13.9)	61 452 (18.5)
GLP‐1RA ± OAD, n (%)	27 481 (6.2)	16 430 (5.0)
Insulin + GLP‐1RA ± OAD, n (%)	13 095 (3.0)	9805 (3.0)
Any GLP‐1RA use, n (%)	40 576 (9.2)	26 235 (7.9)
Exenatide	3202 (7.9)	2260 (8.6)
Exenatide ER	10 291 (25.4)	6358 (24.2)
Albiglutide	2086 (5.1)	1240 (4.7)
Dulaglutide	5174 (12.8)	3169 (12.1)
Liraglutide	23 006 (56.7)	15 009 (57.2)
Any SGLT2i use, n (%)	51 997 (11.8)	29 103 (8.8)
Canagliflozin	35 891 (69.0)	20 350 (69.9)
Dapagliflozin	11 170 (21.5)	5836 (20.1)
Empagliflozin	6530 (12.6)	3791 (13.0)

Note

All data are presented as n (%).

Abbreviations: ER, extended release; GLP‐1, glucagon‐like peptide‐1 receptor agonist; OAD, oral antidiabetes drug; SGLT2i, sodium‐glucose co‐transporter‐2 inhibitor.

Overall, the use of GLP‐1RAs and SGLT2is was low (<12% of patients) and slightly lower in the ASCVD group compared to the non‐ASCVD group. Liraglutide and canagliflozin were the most prevalent GLP‐1RA and SGLT2i agents, respectively. Insulin use was more prevalent in the ASCVD cohort vs the non‐ASCVD cohort (18.5% vs 13.9%).

### 5.3 Healthcare specialist visits

A low and similar proportion of patients visited an endocrinologist during 2015, regardless of ASCVD status (8.0% of those non‐ASCVD; 8.7% of those with ASCVD). In the ASCVD group, 40% had visited a cardiologist during 2015, compared to 11% in the non‐ASCVD group.

### 5.4 Subgroup analysis by age category

**Figure 1:**
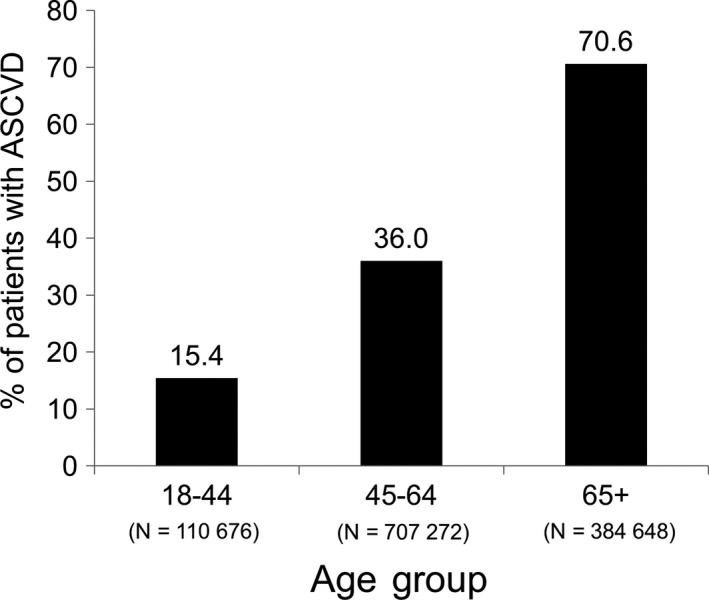
Prevalence of atherosclerotic cardiovascular disease (ASCVD) among 1 202 596 patients with T2DM within age subgroups

The prevalence of ASCVD increased with increasing age category Figure 1. In each age category, all diabetes‐related complications included in the analysis were present at a higher prevalence among the ASCVD cohorts as compared to the non‐ASCVD cohorts Table 3.

**Table 3:** Prevalence of diabetes‐related complications by atherosclerotic cardiovascular disease (ASCVD) status and age category in a real‐world 2015 population with type 2 diabetes

Diabetes‐related complications, n (%)	All Patients	ASCVD status		% difference, ASCVD vs Non‐ASCVD
		Non‐ASCVD	ASCVD	
**Total study population**				
	**N = 1 202 596 (100.0%)**	**n = 659 498 (54.8%)**	**n = 543 938 (45.2%)**	
Hypertension	950 941 (79.1)	472 299 (71.6)	478 642 (88.1)	+16.5%
Dyslipidemia	934 967 (77.8)	484 175 (73.4)	450 792 (83.0)	+9.6%
Retinopathy	145 528 (12.1)	63 101 (9.6)	82 427 (15.2)	+5.6%
Nephropathy	183 043 (15.2)	61 975 (9.4)	121 068 (22.3)	+12.9%
Cerebrovascular	118 557 (9.9)	0 (0.0)	118 557 (21.8)	+21.8%
Cardiovascular^a^	334 933 (27.9)	23 545 (3.6)	311 388 (57.3)	+53.7%
Peripheral vascular^b^	125 519 (10.4)	14 388 (2.2)	111 131 (20.5)	+18.3%
Metabolic	149 080 (12.4)	79 359 (12.0)	69 721 (12.8)	+0.8%
**18‐44 y**				
	**N = 110 676 (100.0%)**	**n = 93 646 (84.6%)**	**n = 17 030 (15.4%)**	
Hypertension	58 849 (53.2)	47 530 (50.8)	11 319 (66.5)	+15.7%
Dyslipidemia	63 249 (57.2)	52 095 (55.6)	11 154 (65.5)	+9.9%
Retinopathy	5756 (5.2)	4577 (4.9)	1179 (6.9)	+2.0%
Nephropathy	6505 (5.9)	4815 (5.1)	1690 (9.9)	+4.8%
Cerebrovascular	2079 (1.9)	0 (0.0)	2079 (12.2)	+12.2%
Cardiovascular^a^	6829 (6.2)	1267 (1.4)	5562 (32.7)	+31.3%
Peripheral vascular^b^	3110 (2.8)	1272 (1.4)	1838 (10.8)	+9.4%
Metabolic^c^	12 983 (11.7)	10 838 (11.6)	2145 (12.6)	+1.0%
**45‐64 y**				
	**N = 707 272 (100.0%)**	**n = 452 819 (64.0%)**	**n = 254 453 (36.0%)**	
Hypertension	550 626 (77.9)	332 391 (73.4)	218 325 (85.8)	+12.4%
Dyslipidemia	558 550 (79.0)	334 869 (76.2)	213 681 (84.0)	+7.8%
Retinopathy	73 045 (10.3)	41 821 (9.2)	31 224 (12.3)	+3.1%
Nephropathy	77 713 (11.0)	38 517 (8.5)	39 196 (15.4)	+6.9%
Cerebrovascular	40 967 (5.8)	0 (0.0)	40 967 (16.1)	+16.1%
Cardiovascular^a^	137 152 (19.4)	12 704 (2.8)	124 448 (48.9)	+46.1%
Peripheral vascular^b^	46 365 (6.6)	8728 (1.9)	37 637 (14.8)	+12.9%
Metabolic^c^	94 700 (13.4)	57 490 (12.7)	37 210 (14.6)	+1.9%
**≥65 y**				
	**N = 384 648 (100.0%)**	**n = 113 033 (29.4%)**	**n = 271 615 (70.6%)**	
Hypertension	341 466 (88.8)	92 378 (81.7)	249 088 (91.7)	+10.0%
Dyslipidemia	313 168 (81.4)	87 211 (77.2)	225 957 (83.2)	+6.0%
Retinopathy	66 727 (17.4)	16 703 (14.8)	50 024 (18.4)	+3.6%
Nephropathy	98 825 (25.7)	18 643 (16.5)	80 182 (29.5)	+13.0%
Cerebrovascular	75 511 (19.6)	0 (0.0)	75 511 (27.8)	+27.8%
Cardiovascular^a^	190 952 (49.6)	9574 (8.5)	181 378 (66.8)	+58.3%
Peripheral vascular^b^	76 044 (19.8)	4388 (3.9)	71 656 (26.4)	+22.5%
Metabolic^c^	41 397 (10.8)	11 031 (9.8)	30 366 (11.2)	+1.4%

a Category includes any cardiovascular complication, not limited to those used to define “ASCVD” (acute coronary syndrome, history of myocardial infarction, angina pectoris, peripheral arterial disease presumed to be of atherosclerotic origin, transient ischaemic attack and coronary or other arterial revascularization).

b Category includes any peripheral vascular disease, not limited to “peripheral arterial disease presumed to be of atherosclerotic origin” which was part of the “ASCVD” definition.

c Category includes ketoacidosis, hyperosmolar and “other coma.”

**Figure 2:**
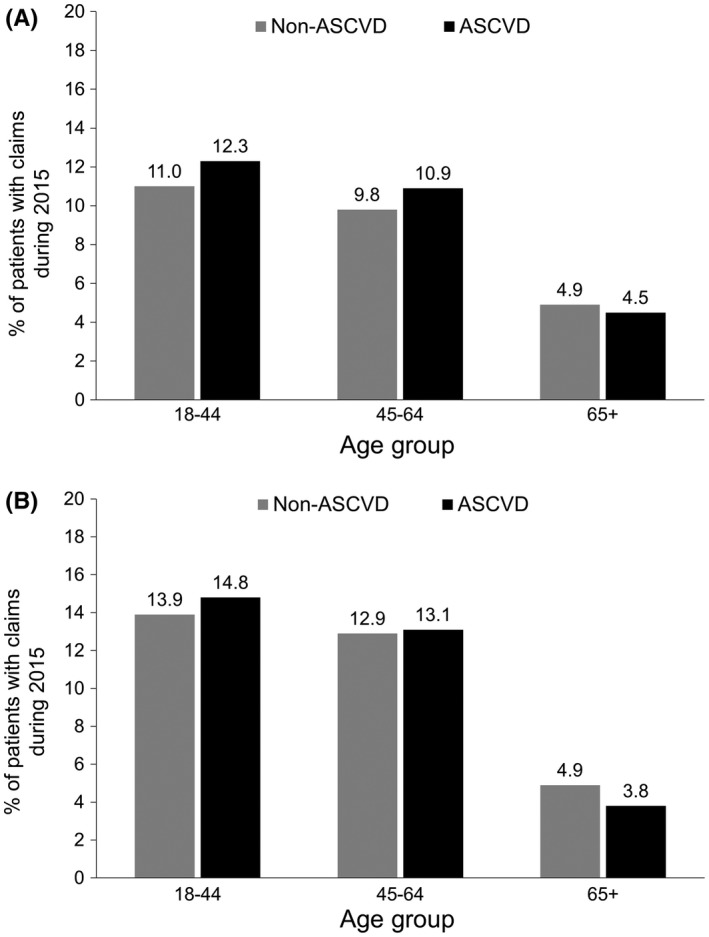
Percentage of patients with T2DM using A, GLP‐1RAs and B, SGLT2is during 2015 by age and atherosclerotic cardiovascular disease (ASCVD) status. Abbreviations: ASCVD, atherosclerotic cardiovascular disease; GLP‐1RA, glucagon‐like peptide‐1 receptor agonist; SGLT2i, sodium‐glucose co‐transporter‐2 inhibitor

The proportion of patients that used GLP‐1RA or SGLT2i agents was 5% or lower among the ≥65 age subgroup, regardless of ASCVD status Figure 2.

## 6 DISCUSSION

In this cross‐sectional analysis of data from a large national US administrative claims database, 45.2% of 1 202 596 patients with T2DM had ASCVD identified based on ICD codes. The ASCVD group was older, on average, and had a higher percentage of patients with Medicare insurance. During 2015, the proportion of patients using GLP‐1RA and SGLT‐2i agents was very low overall in this population (<11%), even among individuals with ASCVD (<9%). Less than 9% of patients with T2DM had visited an endocrinologist, regardless of ASCVD status, which may indicate that a large percentage of diabetes care is being provided by primary care physicians. Among patients with T2DM and ASCVD, 40% had seen a cardiologist during 2015.

Findings from the current analysis are consistent with those of a recent similar cross‐sectional analysis based on electronic health record data from Cleveland Clinic (Ohio and Florida) for the year 2016.^12^ The Cleveland Clinic study included data from 96 569 individuals with T2DM and reported that 42.8% had CVD, which is highly consistent with the proportion with established ASCVD in the current study. In the total Cleveland Clinic study population, usage of GLP‐1RAs and SGLT2is was each <5.5%, and even lower in the ASCVD group. The majority of patients (>80%) had not had an endocrinology visit in the past year.^12^

As previously noted, improved cardiovascular outcomes in patients with T2DM and CVD treated with empagliflozin and liraglutide were demonstrated in the EMPA‐REG‐OUTCOME and LEADER trials, respectively,^8^, ^9^ which led to the ADA recommending empagliflozin and liraglutide in patients with long‐standing sub‐optimally controlled T2DM and ASCVD in 2017.^4^ Additional CV outcomes data with the use of GLP‐1RA and SGLT2i drug classes in patients with T2DM have shown benefits with the SGLT2i, canagliflozin,^15^ and the GLP‐1RA, semaglutide.^16^ The Canagliflozin Cardiovascular Assessment Study (CANVAS) programme combined data from two randomized trials (N = 10 142) and found that canagliflozin significantly reduced the composite CV outcome (death from CV causes, nonfatal MI or nonfatal stroke) compared with placebo in participants with T2DM who had high CV risk (HR 0.86 [95% CI 0.75‐0.97]).^15^ In SUSTAIN‐6, a double‐blind, randomized trial (N = 3297), semaglutide significantly reduced the composite primary outcome (death from CV causes, nonfatal MI or nonfatal stroke) compared with placebo in patients with T2DM and additional established CV risk factors (HR = 0.74, 95% CI = 0.58‐0.95).^16^ Large randomized placebo‐controlled studies with the short‐acting GLP‐1RAs exenatide and lixisenatide have not shown significant reductions in CV outcomes nor CV harm, but rather have reported a neutral effect on CV outcomes.^17^, ^18^ The recently completed REWIND trial was a double‐blind, randomized, placebo‐controlled trial that evaluated the impact of the once‐weekly GLP‐1RA dulaglutide when added to standard of care on CV outcomes in patients with T2D, a majority of patients in the study (69%) did not have established CVD at baseline.^19^ At the time of this manuscript writing, results of the REWIND trial had not yet been formally published, but a press release in November 2018 indicated that dulaglutide 1.5 mg once weekly was associated with a reduction in MACE “across a broad range of people with type 2 diabetes.”^20^

Collectively, available CV outcomes data support the use of certain GLP‐1RA and SGLT2i agents in patients with T2DM who are at high risk for or have established ASCVD. The 2018 ADA Standards of Medical Care recommendations state that “for patients with type 2 diabetes who have ASCVD, on lifestyle and metformin therapy, it is recommended to incorporate an agent with strong evidence for cardiovascular risk reduction, especially those with proven benefit on both major adverse cardiovascular events and cardiovascular death, after consideration of drug‐specific patient factors.”

A limitation of the current analysis is the reliance on ICD‐9/‐10 codes alone to document ASCVD and comorbidities, since these codes may be impacted by provider coding practices and subject to coding error. Also, substantial differences in insurance coverage patterns observed between the groups could potentially impact drug and healthcare resource utilization patterns: the non‐ASCVD group had a much higher proportion of commercially insured patients, whereas approximately half the ASCVD group were covered by Medicare. Another potential limitation is that patients may have had visits to endocrinologists and/or cardiologists closely adjacent to the 1‐year study period window, and therefore not been captured in the assessment of utilization. Further, it was not possible to determine the type of provider who prescribed the antidiabetes therapies. In addition, these data did not include uninsured patients and thus may not be entirely generalizable. Nonetheless, the large number of patients (more than one‐half‐million in each cohort) that were included, and the nationwide sampling, allow for a certain degree of generalizability of these findings.

Although these cross‐sectional data do not capture nuances of clinical practice, they do serve as a useful tool for capturing the prevalence of medication use and overall care patterns in a large cohort of patients with T2DM and ASCVD.

## 7 CONCLUSIONS

This analysis of a large, real‐world claims database showed a high prevalence of ASCVD among T2DM patients, particularly among patients 65 years of age and older. The findings confirmed, as a baseline assessment, low usage of GLP‐1RA and SGLT2i agents in these at‐risk patients during 2015, prior to release of the first ADA guidelines (2017) to recommend use of these agents in patients with ASCVD. Future analyses will be of interest to assess for changes in the use of GLP‐1RAs and SGLT2is pursuant to updated ADA and EASD recommendations regarding benefits of these agents to patients with T2DM and ASCVD.

## CONFLICT OF INTEREST

W. Weng, R. Ganguly, M. Hersloev, J. Brett and T. Hobbs are current employees of Novo Nordisk Inc., the funding body for this study. Y. Tian was a contracted employee of Novo Nordisk Inc., the funding body for this study, during study conduct. S. X. Kong was an employee of Novo Nordisk, Inc., during study conduct.

## AUTHORS’ CONTRIBUTIONS

W. Weng, Y. Tian, S. X. Kong, R. Ganguly and T. Hobbs made substantial contributors to conception and design, or acquisition of data, or analysis and interpretation of data. All others were involved in drafting the manuscript or revising it critically for important intellection content, gave final approval of the version to be published and agree to be accountable for all aspects of the work in ensuring that questions related to the accuracy or integrity of any part of the work are appropriately investigated and resolved.

## MEDICAL WRITING ASSISTANCE

Writing assistance was provided by Kulvinder Katie Singh, PharmD, through Churchill Communications (Maplewood, NJ) and funded by Novo Nordisk, Inc.

## ETHICS STATEMENT

This was a retrospective, noninterventional study using data from the IBM MarketScan database which contains de‐identified patient claims data. The IBM MarketScan database is fully compliant with the Health Insurance Portability and Accountability Act of 1996.

## Data Availability

The data that support the findings of this study are available from IBM/Truven. Restrictions apply to the availability of these data, which were used under licence for this study. Data are available Wayne Weng with the permission of IBM/Truven.
