# Supplementary material for: The prevalence of cardiovascular disease and antidiabetes treatment characteristics among a large type 2 diabetes population in the United States
Source: Endocrinol Diabetes Metab. 2019 May 22;2(3):e00076. doi: 10.1002/edm2.76 (PMC6613222; doi:10.1002/edm2.76)
Supplement: Supplementary file 1 [file EDM2-2-e00076-s001.docx]

**Supplemental Table S1.** A complete list of all ICD-9/ICD-10 codes used to define/identify established ASCVD

| **ADA Class** | **ICD-9** |  | **ICD-10** |  |
| --- | --- | --- | --- | --- |
|  | **430 - 438** | **Cerebrovascular Disease** | **I60 - I69** | **Cerebrovascular Diseases** |
| Stroke | 430 | Subarachnoid hemorrhage | I60 | Nontraumatic subarachnoid hemorrhage |
| Stroke | 431 | Intracerebral hemorrhage | I61 | Nontraumatic intracerebral hemorrhage |
| Stroke | 432 | Other and unspecified intracranial hemorrhage | I62 | Other and unspecified nontraumatic intracranial hemorrhage |
| Stroke | 433 | Occlusion and stenosis of precerebral arteries | I63 | Cerebral infarction |
| Stroke | 434 | Occlusion of cerebral arteries | I65 | Occlusion and stenosis of precerebral arteries, not resulting in cerebral infarction |
| TIA | 435 | Transient cerebral ischemia |  |  |
| Stroke | 436 | Acute, but ill-defined, cerebrovascular disease | I66 | Occlusion and stenosis of cerebral arteries, not resulting in cerebral infarction |
| Stroke | 437.0 | Cerebral atherosclerosis | I67.2 | Cerebral atherosclerosis |
| Stroke | 437.1 | Other generalized ischemic cerebrovascular disease | I67.81  I67.82  I67.83  I67.84 | Acute cerebrovascular insufficiency  Cerebral ischemia  Posterior reversible encephalopathy syndrome  Cerebral vasospasm and vasoconstriction |
| Stroke | 438 | Late effects of cerebrovascular disease | I69 | Sequelae of cerebrovascular disease |
| Stroke |  |  | R29.7 | National Institutes of Health Stroke Scale (NIHSS) score  **Exclude R29.700 |
|  | **410 - 414** | **Ischemic Heart Disease** | **I20 - I25** | **Ischemic Heart Diseases** |
| MI | 410 | Acute myocardial infarction | I21 | ST elevation (STEMI) and non-ST elevation (NSTEMI) myocardial infarction |
| MI |  |  | I22 | Subsequent ST elevation (STEMI) and non-ST elevation (NSTEMI) myocardial infarction |
| ACS | 411 | Other acute and subacute forms of ischemic heart disease | I24 | Other acute ischemic heart diseases |
| MI | 412 | Old myocardial infarction | I23 | Certain current complications following ST elevation (STEMI) and non-ST elevation (NSTEMI) myocardial infarction (within the 28 day period) |
| Angina | 413 | Angina pectoris | I20 | Angina pectoris |
| ACS | 414 | Other forms of chronic ischemic heart disease  **Exclude 414.1 | I25 | Chronic ischemic heart disease    **Exclude I25.3 & I25.4 |
| ACS | V71.7 | Observation for suspected cardiovascular disease |  |  |
|  | **440 - 449** | **Diseases of Arteries, Arterioles, and Capillaries** | **I70 - I79** | **Diseases of Arteries, Arterioles, and Capillaries** |
| PAD | 440 | Atherosclerosis | I70 | Atherosclerosis |
| PAD | 443.8 | Other specified peripheral vascular diseases |  |  |
| PAD | 443.9 | Peripheral vascular disease, unspecified | I73.9 | Peripheral vascular disease, unspecified |
| PAD | 444 | Arterial embolism and thrombosis | I74 | Arterial embolism and thrombosis |
| PAD | 445 | Atheroembolism | I75 | Atheroembolism |
|  |  | **Other CV-related** |  | **Other CV-related** |
| MI | 429.7 | Certain sequelae of myocardial infarction, not elsewhere classified |  |  |
| TIA |  |  | G45 | Transient cerebral ischemic attacks and related syndromes |
| PAD | 459.8 | Other specified disorders of circulatory system | I99 | Other and unspecified disorders of circulatory system |
| PAD  PAD | 459.9 | Unspecified circulatory system disorder | I99  Z86.7 | Other and unspecified disorders of circulatory system  Personal history of diseases of the circulatory system |
|  | V12.5 | Personal history of diseases of circulatory system |  |  |
| Revascularization* | V45.81 | Aortocoronary bypass status | Z98.6 | Angioplasty status |
| Revascularization* | V45.82 | Percutaneous transluminal coronary angioplasty status | Z98.6  Z95.1 | Angioplasty status  Presence of cardiac and vascular implants and grafts |
| Revascularization* | V15.1 | Personal history of surgery to heart and great vessels, presenting hazards to health | Z95.5 | Presence of coronary angioplasty implant and graft |
| Revascularization* |  |  | Z95.8 | Presence of other cardiac and vascular implants and grafts |
| Revascularization* |  |  | Z95.9 | Presence of cardiac and vascular implant and graft, unspecified |
